# Effects of maternal epidural analgesia on the neonate - a prospective cohort study

**DOI:** 10.1186/s13052-014-0099-x

**Published:** 2014-12-10

**Authors:** Bikash Shrestha, Amit Devgan, Mukti Sharma

**Affiliations:** Department of Pediatrics, Nepalese Army Institute of Health Sciences, Shree Birendra Hospital, Swayambhu, Chhauni, Kathmandu, 44620 Nepal; Department of Pediatrics, Armed Forces Medical College, Pune, 411040 India

**Keywords:** Birth asphyxia, Breast feeding, Epidural analgesia, Neonate, Newborns

## Abstract

**Background:**

Epidural analgesia is one of the most popular modes of analgesia for child birth. There are controversies regarding adverse effects and safety of epidural analgesia. This study was conducted to study the immediate effects of the maternal epidural analgesia on the neonate during early neonatal phase.

**Methods:**

A prospective cohort study of 100 neonates born to mothers administered epidural analgesia were compared with 100 neonates born to mothers not administered epidural analgesia in terms of passage of urine, initiation of breast feeding, birth asphyxia and incidence of instrumentation.

**Results:**

There was significant difference among the two groups in the passage of urine (P value 0.002) and incidence of instrumentation (P value 0.010) but there was no significant difference in regards to initiation of breast feeding and birth asphyxia.

**Conclusions:**

Epidural analgesia does not have any effect on the newborns in regards to breast feeding and birth asphyxia but did have effects like delayed passage of urine and increased incidence of instrumentation.

## Background

Safe neonatal outcome is the ultimate aim of any delivery. Pain management is a major issue and part of normal labours. Among various modes of pain management, epidural analgesia is considered a very safe and popular mode of analgesia for child birth [1-3]. Considering use of various types of analgesia, it would be desirable to know the adverse effects of any analgesia being used. Epidural analgesia is one of the extensively studied modes of analgesia in labours. Most studies which have been conducted over epidural analgesia primarily focus on the maternal parameters [3-6]. Despite its popularity, epidural analgesia has remained controversial in regards to its safety [4-6]. The meta-analysis regarding the safety of epidural analgesia has remained inconclusive [7,8]. Considering the controversial aspects of epidural analgesia, we intended to study the immediate effects of epidural analgesia in the newborns born to mothers with epidural analgesia and compare with the newborns born to mothers without epidural analgesia.

## Methods and methodology

### Methods

100 consecutive mothers who were given epidural analgesia and 100 mothers who were not given epidural analgesia for normal labours were enrolled into the study. The neonates born to two groups of mothers were compared in regards to the time of passage of urine, the initiation of onset of breast feeding, birth asphyxia and instrumentation in the form of vacuum or forceps delivery.

### Inclusion and exclusion criteria

The mothers who were regularly followed up in our antenatal clinic were included in the study after taking the informed consent. Caesarean sections, preterms (Less than 37 weeks of completed gestation), low birth weights (Less than 2.5 kg), antenatally detected major congenital anomalies, multiple gestations, high risk antenatal factors like gestational diabetes, pregnancy induced hypertension, recurrent abortions, elderly primigravida (Above 40 years) and those who did not provide consent were excluded from the study.

### Study design, sample size and place of study

Incidence of epidural analgesia is around 3-5% of all labours in our institute. Considering the delivery rate of around 3000 per year, 100 cases each of epidural analgesia and 100 controls without epidural analgesia was determined sample size. The prospective cohort study was conducted in a tertiary care teaching hospital in India between Jan 2012 and Jan 2013. The study was approved by the institutional review board committee of Maharashtra University of Health Sciences, Nashik, India.

### Methodology

Epidural analgesia is given voluntarily to the normal delivery cases in our institute. Informed written consent was taken from the participants. The pregnant ladies who demanded analgesia for labour pain were provided with epidural analgesia, consisting of 10 ml of 0.125% bupivacaine & 20 mcg fentanyl. The neonates born were followed up to 3 days to note the various study parameters including passage of urine, onset of breast feeding, birth asphyxia and instrumental interventions if any. The study performa were filled up by the duty resident every day during the morning and evening rounds. During the same period, neonates who were born to mothers without epidural analgesia were also followed up and various parameters noted. The results were compared among the two groups and statistical analysis was done using the software Epi Info 3.5.1 and P value was calculated by Chi square test and Fisher’s exact test. P value of <0.05 was considered as statistically significant.

## Results

Table 1 represents the comparative baseline maternal demographic data of the two groups in regards to parity, age group, the addresses and the religions. The baseline maternal data between the two groups are non significant. Table 2 represents the sexes and the different weight groups of newborns in the two groups which were comparable to each other.

**Table 1:** **Baseline maternal demographic data compared between the epidural and non epidural groups**

	**Cases**	**Control**	**P value**
**Parity**			
Primi	59	47	0.118
Multi	41	53	
**Age group**			
< 20 years	9	4	
20-30 years	89	92	0.267
> 30 years	2	4	
**Address**			
Within same district	82	91	0.096
Out of same district	18	9	
**Religion**			
Hindu	88	85	
Muslim	11	12	0.578
Christian	1	3

**Table 2:** **The sex and weight groups of the newborns in the epidural and non epidural groups**

	**Cases**	**Control**	**P value**
**Sex**			
Male	53	47	0.479
Female	47	53	
**Birth weight group**			
2.5-3 kg	60	69	
3-3.5 kg	37	28	0.391
3.5-4 kg	3	3	

The timing of passage of urine by the newborns that were born with and without epidural analgesia has been represented in Table 3. The passage of urine in first six hours, then between six and 24 hours and more than 24 hours were noted. 42 newborns in epidural analgesia group and 58 newborns in non epidural analgesia group had passed urine in the first 6 hours. In the six-24 hours group, there were 49 newborns in epidural group and 42 in non epidural group. There were total 9 newborns that passed urine beyond 24 hours and all of them were in epidural analgesia group. The P value was highly significant among the two groups (P value-0.002). Thus, the results have shown that in newborns born to mothers with epidural analgesia, there is higher tendency to pass urine later than the newborns without epidural analgesia.

**Table 3:** **The timing of passage of urine in the epidural and non epidural groups**

**Time of passage of urine**	**Cases**	**Control**	**Total**	**P value**
**0-6 hours**	42	58	110	
**6-24 hours**	49	42	91	0.002
**>24 hours**	9	0	9	
**Total**	100	100	200	

The timing of initiation of breast feeding among the newborns those were born to mothers with and without epidural analgesia is shown in Table 4. The timing of breast feeding was divided into 3 groups, 0-six hours, six-24 hours and more than 24 hours. In epidural group and non epidural group, there were 96 and 98 newborns each who had established breast feeding successfully within six hours. Only one newborn in both the groups had established breast feeding between six-24 hours. In epidural group, there were three cases and in non epidural analgesia, there was only one case where breast feeding was established after 24 hours. The P value among the two groups was not significant (P value 0.60).

**Table 4:** **The timing of initiation of breast feeding in the epidural and non epidural groups**

**Time of onset of breast feeding**	**Cases**	**Control**	**Total**	**P value**
**0-6 hours**	96	98	194	
**6-24 hours**	1	1	2	0.60
**>24 hours**	3	1	4	
**Total**	100	100	200	

The number of birth asphyxia which has occurred are tabulated in Table 5. In epidural analgesia group, three had birth asphyxia and in non epidural analgesia, only one had birth asphyxia. Although higher number of birth asphyxias had occurred in epidural group, it was not statistically significant (P value 0.621).

**Table 5:** **The number of birth asphyxias in the epidural and non epidural groups**

**Birth asphyxia**	**Cases**	**Control**	**Total**	**P value**
**Yes**	3	1	4	
**No**	97	99	196	0.621
**Total**	100	100	200	

The number of instrumental deliveries which had taken place in the two groups has been depicted in Table 6. Of the total 13 instrumental deliveries, which included both vacuum and forceps, 11 were from epidural analgesia group and only two were from non epidural analgesia group. The result in the two groups was highly significant (P value 0.010).

**Table 6:** **The number of instrumental deliveries in the epidural and non epidural groups**

**Instrumentation**	**Cases**	**Control**	**Total**	**P value**
**Yes**	11	2	13	
**No**	89	98	187	0.010
**Total**	100	100	200	

## Discussion

We had studied the various parameters in the newborns born to epidural analgesia group and compared with the newborns born to mothers without epidural analgesia. The results included the timing of passage of first urine, onset of breast feeding, birth asphyxia and instrumental delivery.

The timing of passage of urine had been divided into 3 groups, a) within first six hours, b) between six & 24 hours and c) more than 24 hours. In the study, lesser numbers of newborns had passed urine within the first six hours in epidural analgesia group (42 out of 110 or 38.2%) than non epidural analgesia group (58 out of 110 or 52.8%), whereas higher number of neonates had passed urine after six hours in the epidural analgesia group (49 out of 91 or 53.8%) than in non epidural group (42 out of 91 or 46.2%). Among nine newborns who had passed urine after 24 hours only, all were in epidural analgesia group. Although the passage of urine has been delayed, it was within physiological period of 48 hours. The delay in passage of urine was highly significant among the newborns in epidural analgesia group (P value 0.002). Epidural analgesia is known to cause urinary retention in the mothers post partum due to the effects of fentanyl [3,6,7,9,10]. Most studies are unable to explain the exact mechanism of post partum urinary retention in the mothers with epidural analgesia. There has been no documentation in the literature regarding the urinary retention in newborns born to mothers with epidural analgesia. This study is the first one to report this finding. Probably, the maternal drug transferred to the newborn could have led to urinary retention as we had used fentanyl in the mothers for epidural analgesia. However, urinary parameters in the mother were not noted in our study.

Difficulty in establishment of breast feeding is another controversial issue in the field of epidural analgesia. Successful breast feeding is one of the aims of successful labours. In our study, majority of the mothers had initiated and established breast feeding within six hours of birth confidently (194 out of 200 or 97%). Only six babies had delayed onset of breastfeeding, because of the birth asphyxia and other medical conditions for which oral feeds were withheld. There was no significant difference among the cases and the control groups with P value being 0.60. Various studies have reported that epidural analgesia may lead to difficulty in establishing early breast feeding [11,12]. There are other studies which refute such relationship [1,13-15]. Epidural analgesia per se should not have bearing upon the initiation of breast feeding in the newborns, unless the overdosing of the analgesia may make the mother feel drowsy and lead to delay in the establishment of breast feeding. Considering the appropriate dose of epidural analgesia, there should be no effect upon the initiation of the breast feeding as elicited in the study.

Epidural analgesia has also been implicated in being associated with prolonged labour, respiratory distress and lower APGAR scores in the neonates [16-19]. At the same time, there are other studies which do not support such association [1-3,20,21]. In our study, we had studied the incidence of birth asphyxia in the epidural and non epidural groups. We had considered APGAR less than six at five minutes as birth asphyxia. In epidural analgesia group, three babies had birth asphyxia and in non epidural group, only one baby had suffered birth asphyxia. However, the difference was not statistically significant (P value 0.621). Epidural analgesia may be implicated in prolonging the labors by reducing the pain and thus reducing bearing down efforts, however, it is not directly contributing to birth asphyxia per se.

Another controversial aspect of epidural analgesia which we intended to study was the higher number of instrumental deliveries associated with epidural analgesia. There are studies which claim higher incidence of instrumental deliveries, including Caesarean delivery with epidural analgesia [19-23]. There are other studies which do not show such relationship [1,2,24]. In our study, Caesarean section was excluded and we could not comment upon the incidence of Caesarean section. In our study total 13 cases required instrumental interventions, including both forceps and vacuum. Out of these, 11 were from epidural analgesia group and only two from non epidural group. The difference in the two groups in the study has been statistically significant (P value 0.010). This may perhaps be explained by the poorer bearing down efforts in the mothers with epidural analgesia. At the same time, it should also be acknowledged that more complicated pregnancies are more likely to be assisted with analgesia and thus may end up being intervened more with instrumental deliveries. Confounding factors like prolonged second stage, dystocias, delayed pushing, more complicated pregnancies, parity, age, may perhaps explain the difference.

## Summary and conclusions

A prospective cohort study was conducted to study the effects of maternal epidural analgesia on the neonate during early neonatal phase. 100 newborns born to mothers who were administered epidural analgesia and 100 newborns born to mothers who were not given epidural analgesia were compared among various study parameters. The newborns born to mothers with epidural analgesia tended to pass urine later significantly than the non epidural group. There was significantly increased incidence of instrumental deliveries in epidural group than in non epidural group. However, there have been no immediate effects upon breast feeding and birth asphyxia in our study. The effect of epidural analgesia on the neonate is of immense significance and should be further explored in the future with more elaborate randomized controlled multi-centre studies.
